# Changes in Human Milk Fatty Acid Composition during Lactation: The Ulm SPATZ Health Study

**DOI:** 10.3390/nu11122842

**Published:** 2019-11-20

**Authors:** Linda P. Siziba, Leonie Lorenz, Bernd Stahl, Marko Mank, Tamas Marosvölgyi, Tamas Decsi, Dietrich Rothenbacher, Jon Genuneit

**Affiliations:** 1 Pediatric Epidemiology, Department of Pediatrics, University Medicine Leipzig, 04103 Leipzig, Germany; Linda.Siziba@medizin.uni-leipzig.de; 2 Institute of Epidemiology and Medical Biometry, Ulm University, 89075 Ulm, Germany; leonie.lorenz1@gmx.de (L.L.); Dietrich.Rothenbacher@uni-ulm.de (D.R.); 3 Danone Nutricia Research, 3584 CT Utrecht, The Netherlands; Bernd.Stahl@danone.com (B.S.); Marko.Mank@danone.com (M.M.); 4 Department of Chemical Biology & Drug Discovery, Utrecht Institute for Pharmaceutical Sciences, Utrecht University, 3584 Utrecht, The Netherlands; 5 Department of Paediatrics, Medical School, University of Pécs, 7623 Pécs, Hungary; marosvolgyi.tamas@pte.hu (T.M.); decsi.tamas@pte.hu (T.D.)

**Keywords:** human milk, fatty acids, lactation, composition

## Abstract

The lipid fraction of human milk provides the infant with the fatty acids that are necessary for optimal growth and development. The aim of this study was to investigate the fatty acid composition of human milk at three time points during lactation and its change over time using appropriate statistical methods. Human milk samples from breastfeeding mothers at 6 weeks (*n* = 706), 6 months (*n* = 483), and 12 months (*n* = 81 with all three time points) were analyzed. Centered log-ratio (clr) transformation was applied to the fatty acid data. Principal component analysis (PCA) and generalized linear model-based repeated measure analysis were used to assess changes over time. The total lipid content was significantly higher at 6 months (β = 0.199, *p* < 0.029) and 12 months of lactation (β = 0.421, *p* < 0.001). The constituents of C20:3n-6 and C20:3n-3 were lower at 6 months (*p* < 0.001). Four distinct sub-compositional fatty acid groups were only identified at 12 months of lactation. The inclusion of small fatty acids of small constituent size in the analysis resulted in a shift in the balance between fatty acid constituents. Human milk fatty acid composition during prolonged lactation is different from that of human milk during a short duration of lactation. Our findings support the hypothesis that a combination of multiple fatty acids is important in fatty acid profiling beyond the presentation of individual fatty acids. Furthermore, the high variability of small fatty acids warrants attention because a compositional analysis may show more pronounced changes.

## 1. Introduction

Human milk is the ideal source of nutrients for an infant. It is biologically tailored to provide a variety of nutrients, bioactive compounds, and immunological factors, which are crucial for growth and optimal development [1]. Human milk lipids constitute the largest fraction of the total energy intake during infancy, providing an average of 44% energy supply [2]. Thus, the composition of fatty acids supplied through human milk is of great interest because these provide a standard for defining adequate nutrient intakes of infants [3].

Fatty acids in human milk are derived from mobilization of the mother’s endogenous stores, from synthesis in the liver or breast tissue, and from the mother’s diet [4]. Maternal gestational duration, age, stage of lactation, diet, and long-term food habits were shown to be associated with the fatty acid composition of human milk [5,6]. Thus, fatty acid concentrations in human milk will also vary depending on accessibility to food in a specific region [7]. Several studies have reported on the fatty acid composition of human milk in Germany [8,9,10] and other parts of Europe and the world [7,11,12]. However, information on changes in fatty acid composition of human milk as lactation progresses remains controversial. Very few [11,12,13] studies have reported changes in human milk fatty acid composition up to 12 months of lactation. Thus, the important practical question of whether human milk can provide a constant supply of fatty acids for infants even as lactation progresses remains.

It is plausible that the results from human milk fatty acid profiling are controversial because of differences in (1) population characteristics including lifestyle factors, (2) timing of milk sampling post-delivery including colostrum, transitional milk, and mature milk across lactation, (3) statistical methodologies neglecting the compositional nature of the data, and (4) sample sizes. Regarding the compositional nature, fatty acids are often reported individually as a relative measure of the percentage/weight of total fatty acids. However, an increase in the percentage of one fatty acid must result in decreases in the relative percentage of one or more other fatty acids, even when their absolute concentrations remain unchanged [14,15,16]. Thus, no individual component should be interpreted isolated from the others.

Considering this, we investigated the fatty acid composition of human milk at three time points during lactation and its change over time in the Ulm SPATZ Health Study. To overcome the constant sum constraint, we applied centered log-ratio transformation (clr) and used principal component analysis (PCA) to identify groups of fatty acids that are correlated. These fatty acid groups could potentially be more relevant in fatty acid profiling and assessing the effect of time and biological changes in human milk compared to using the traditional groups that are based on chemical structure.

## 2. Materials and Methods 

### 2.1. Study Design and Population

The Ulm SPATZ Health Study is an ongoing birth cohort study in which a total of 1006 newborns and their 970 mothers were recruited from the general population consecutively, during their hospital stay soon after delivery in the University Medical Center Ulm, Southern Germany, between April 2012 and May 2013 [17]. Mothers were excluded if they had inadequate German language skills, outpatient childbirth, maternal age <18 years, postpartum transfer of mother or child to an intensive care unit, or stillbirth. Participation in the study was completely voluntary and informed consent was obtained. Ethical approval was obtained from the Ethics board of Ulm University (No. 311/11).

### 2.2. Data Collection and Measurements

Demographic, lifestyle, and birth-related data including child gender, delivery mode, birth season, birthweight, maternal age, education, parity, pre-pregnancy body mass index (BMI calculated as (mass(kg)/height(m)^2^)), and smoking status (within 1 year prior to delivery) were collected by self-administered questionnaire. Additional information was assessed from electronic hospital charts and from routine screening examinations during pregnancy. Smoking status and alcohol consumption were assessed and defined as previously explained [17]. Human milk samples were collected at approximately 6 weeks, 6 months, and 12 months post-delivery. Mothers were instructed to manually express or pump breast milk between 09:00 h and 12:00 h, after breakfast and before lunch, but at least one hour after the last feeding. In some cases, trained study nurses helped mothers with expressing. Mothers stored the milk in the fridge until study nurses collected it from their homes and delivered it refrigerated to the study center. Additional data were collected at 6 weeks, 6 months, and 12 months post-delivery by telephone interview or postal self-administered questionnaires. Yearly follow-ups of the whole study population are still ongoing.

### 2.3. Fatty Acid Analysis

Human milk samples were stored at −80 °C until analysis of fatty acids between 2015 and 2018 by high-resolution capillary gas-liquid chromatography by using the method of Bligh and Dyer [18] and Beermann et al. [19]. The lipid content was analyzed using the method outlined by Lucas et al. [20] and Jones et al. [21]. Data are reported for 45 fatty acids which include saturated fatty acids (SFAs), monounsaturated fatty acids (MUFAs), trans-fatty acids (TFAs), branched-chain fatty acids (BFAs), and polyunsaturated fatty acids (PUFAs). Peak identification of SFAs, MUFAs, TFAs, and both n-3 and n-6 PUFA methyl esters were verified and calibrated by comparison with authentic standards (NuChek Prep; Elysian, MN, USA: GLC-463, GLC-473, GLC-642, GLC-643, GLC-674, and Sigma-Aldrich Ltd., St. Louis, MO, USA: Supelco PUFA3). The standard mix of BFA methyl esters was originally from Danone Nutricia Research, Utrecht, the Netherlands. Fatty acid concentrations were recorded as %weight of total fatty acids.

### 2.4. Statistical Analysis 

Clr transformations were applied to fatty acid data to account for compositionality. Clr was calculated as the natural log of the quotient of the individual fatty acid concentration over the geometric mean of all fatty acid concentrations within a sample [22]. The geometric mean was used as the denominator as it is a meaningful measure of central tendency for variables with skewed distributions. Principal component analysis (PCA) was used to evaluate correlations between fatty acid prior to generating compositional biplots. Fatty acid groups/clusters were selected based on collinear links of a sub-composition showing a one-dimensional pattern in the compositional biplots for 12 months human milk samples. These fatty acid groups/clusters were calculated by adding the individual crude fatty acid concentrations, after which this sum was clr transformed for inclusion into the analysis models. A general linear model was used to assess the impact of maternal pre-pregnancy BMI, change in weight status between pregnancy and at 6 weeks, age, education, occupation, parity, delivery mode, and gestational age on individual human milk fatty acids. Maternal age was included because of its close association with maternal education, less exposure to cigarette smoking, and (prolonged) breastfeeding [17], thus potentially being an indicator of aspects of life style that might influence human milk fatty acid composition. Additionally, education and occupation were used as proxies for socio-economic status. A general linear model based repeated measure analysis was used to evaluate changes in human milk fatty acid constituents during lactation, adjusting for lifestyle-related factors. Bonferroni adjustment was applied to account for multiple testing. All statistical analyses were performed with SAS version 9.4 (The SAS Institute, Cary, NC, USA).

## 3. Results

Of the 970 mothers enrolled into the Ulm SPATZ Health study, 86% (*n* = 742) mothers were still breastfeeding their infants at 6 weeks. Human milk samples for fatty acid analysis were available from 706 (95.2% of breastfeeding mothers), 483 (87.3% of all breastfeeding mothers), and 83 (52.3% of all breastfeeding mothers) lactating women at 6 weeks, 6 months, and 12 months, respectively. Only 81 mothers provided a human milk sample at all three time points (6 weeks, 6 months, and 12 months). The lactating women were aged 32.7 ± 4.8 years, and the characteristics of those women who provided a human milk sample at any one of the three times points are shown in Table 1.

**Figure 1:**
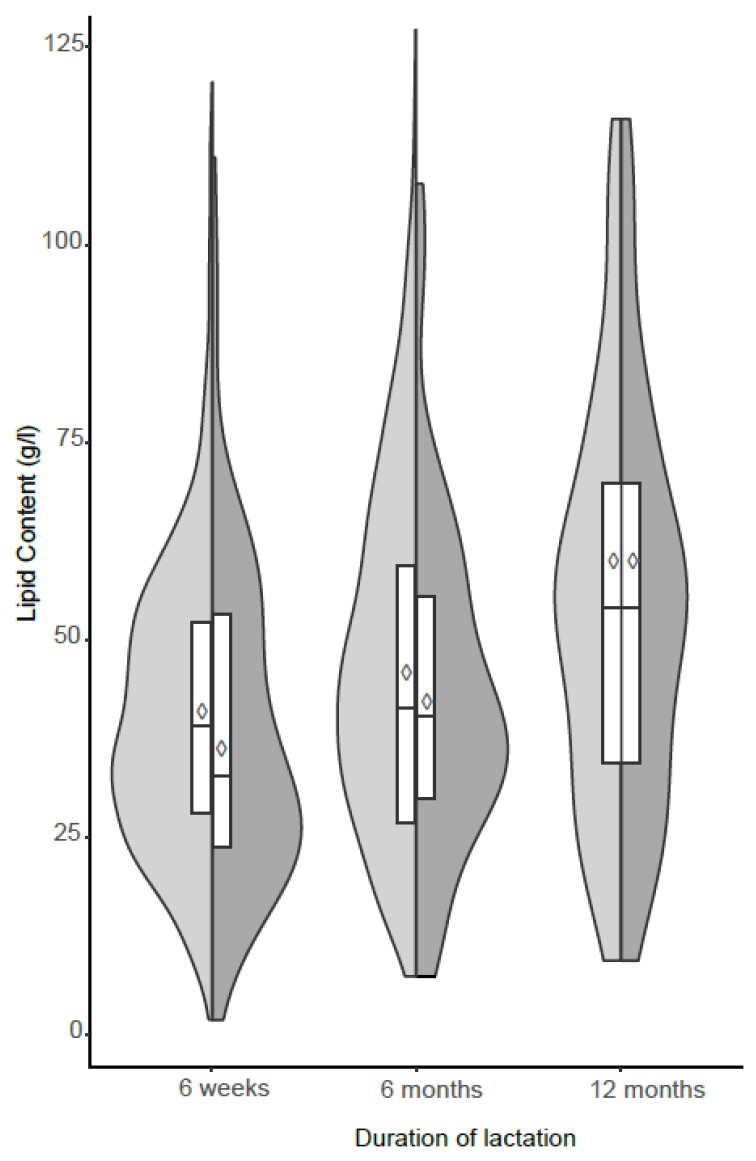
Distribution of total lipid content concentrations (g/L) of human milk samples at 6 weeks, 6 months, and 12 months of lactation. Shaded areas show split violin plots of the density function with inserted boxes indicating the first and third quartile and the median and with inserted ◊ indicating the arithmetic mean. ◊ Arithmetic mean lipid content of given sample si Total sample size 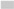 (6 weeks (*n* = 706), 6 months (*n* = 483), 12 months (*n* = 81)), 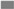 Sub-sampl e size (*n* = 81).

The total lipid content increased significantly at 6 months (β = 0.199, *p* = 0.029) and 12 months (β = 0.421, *p* < 0.001) of lactation (Figure 1). These changes remained significant even after adjusting for education, occupation, parity, gestation period, delivery mode, and maternal age. Results from the Wilcoxon sum rank test showed some significant differences of fatty acid concentrations between 6-week, 6-month, and 12-month samples (Table 2). All MUFAs, BCFAs, and TFAs remained statistically similar during lactation. The constituent sizes of the medium chain fatty acids (MCFAs) lauric acid (C12:0), tridecylic (C13:0), and myristic acid (C14:0), were significantly larger at 6 and 12 months compared to 6 weeks.

Following Bonferroni correction, the n-6 metabolites eicosadienoic (C20:2n-6), dihomo-γ-linolenic acid (DGLA; C20:3n-6) and docosadienoic (C22:2n-6) were statistically lower at 6 months compared to 6 weeks. The n-6 metabolite γ-linolenic acid (GLA; C18:3n-6) was lower at 12 months compared to 6 weeks and 6 months. Dihomo-α-linolenic acid (C20:3n-3) was significantly lower at 6 months, and a decrease of C20:4n-3 at 12 months of lactation was observed.

These differences remained significant when the sample size was restricted to human milk samples measured at 6 weeks and 6 months only (*n* = 467; Supplemental Table S1). Significant differences in the constituent sizes of SFAs, MUFAs, BCFAs, and the essential PUFAs linoleic acid (LA; C18:2n-6), α-linoleic acid (ALA; C18:3n-3), and docosahexaenoic acid (DHA; C22:6n-3) were also observed in this sub-sample.

**Figure 2:**
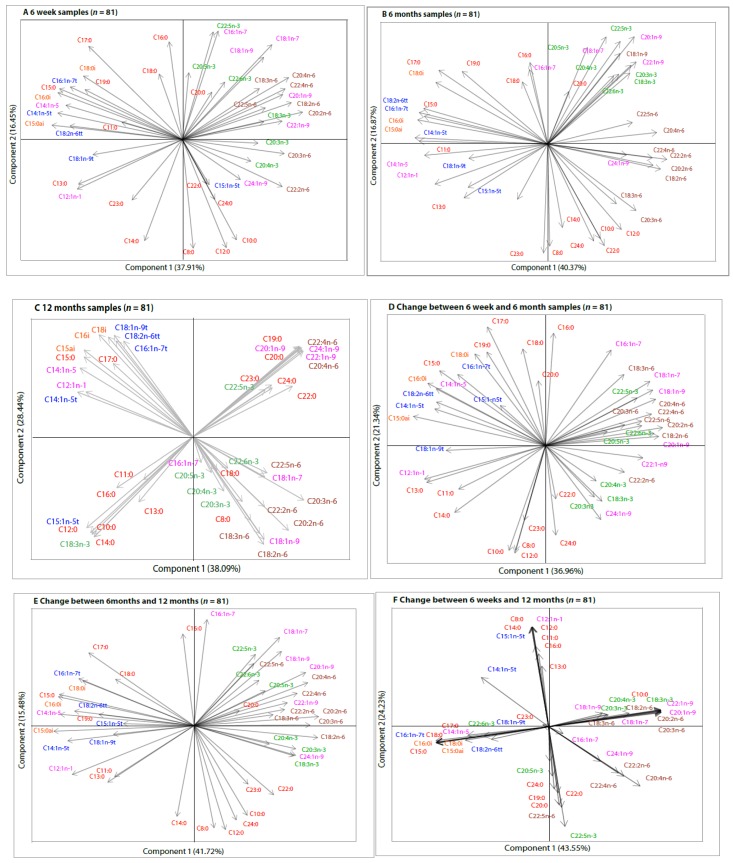
Compositional biplots from principal component analysis (PCA) of centered log-ratio transformed fatty acid concentrations of human milk samples collected at 6 weeks, 6 months, and 12 months (*n* = 81). Colour Key: Red—Saturated fatty acids; Blue—Trans-fatty acids; Green—Omega-3 fatty acids; Pink—Monounsaturated fatty acids; Orange—Branched-chain fatty acids. Compositional biplots of fatty acids analyzed in human milk samples collected at (**A**) 6 weeks; (**B**) 6 months; **(C)** 12 months. Compositional biplots of changes in fatty acid composition between **(D)** 6 weeks and 6 months; **(E)** 6 months and 12 months **(F)** 6 weeks and 12 months.

Compositional biplots obtained from the PCA did not show clear and consistent groupings of fatty acid concentrations of human milk samples collected at 6 weeks and 6 months (Figure 2A,B). This was also the case for the change between 6 weeks and 6 months (Figure 2D). Relative positions between fatty acids remained consistent and there were unclear groupings when all 6 weeks (*n* = 706), all 6 months (*n* = 483) samples, and those who provided samples at both time points (“6 months restricted samples” (*n* = 467)) were analyzed (Supplemental Figure S1).

Compositional biplots of 12 months samples showed that all fatty acids could be combined into at least 1 of four groups based on the correlations and sub-compositions defining a high-variance (Figure 2C). The angles between the individual fatty acids in each sub-compositional group were very small. Despite most of the non-significant changes in individual fatty acid constituents between 6 weeks and 12 months, and 6 months and 12 months (Table 2), the correlations of the changes were quite clear and formed distinct sub-compositions, showing two dimensional patterns (Figure 2E,F).

In a third PCA and compositional biplots, only 32 fatty acids were included. Inclusion of fatty acid into the PCA was based on the most commonly reported fatty acids in previous studies. The compositional biplots from this PCA showed very clear and more distinct patterns of fatty acid sub-compositions based on their correlational properties (Supplemental Figure S2).

The changes in fatty acid concentrations across time were further evaluated using the fatty acid groups derived from the 12 months compositional biplots as well as the traditional chemical groupings (Table 3). In a crude model, the 12 month-derived fatty acid group 2 was significantly higher at 6 months (β = 0.037, *p* = 0.0046) and 12 months (β = 0.114, *p* = <0.0001), group 3 was lower at 12 months (β = −0.059, *p* = 0.0013), and group 4 was lower at 6 months (β = 0.086, *p* = 0.0003) of lactation. These changes remained significant even after adjusting for maternal age, education, occupation, parity, gestation period, delivery mode, BMI, and Bonferroni correction (Table 3). While the relative positions and direction of each of the individual fatty acids changed at each time point in the compositional biplots (Figure 2), the total TFAs, BCFAs, and total PUFAs remained statistically similar at each time point.

## 4. Discussion

In the present birth cohort study, we evaluated the changes in fatty acid composition of human milk sampled at 6 weeks, 6 months, and 12 months post-delivery, using statistical methods accounting for compositional data. Firstly, we observed a marked increase in total lipid content of human milk similar to previous studies [9,12,23,24] and we attribute these changes to the high energy requirements of the growing infant. This increasing proportion of the lipid fraction in human milk provides the infant with adequate energy during the first months of life, when human milk is the sole source of nutrition for the infant. In addition, the average amount of the lipid content in mature milk also changes during the course of a day and increases during an individual feed [25]. On the other hand, our results show that there were two possible distributions among lactating women whose lipid content was available at 6 weeks. Further analysis showed that this distribution was confounded by obesity thus an indication of possible interindividual and intraindividual variations in the lipid content human milk.

In addition, following clr transformation, we observed that palmitic acid (C16:0) remained the major SFA, and oleic acid (C18:1n-9) constituted the largest part of MUFAs. Nonetheless, based on the correlational attributes of fatty acids, our data do not show exclusive and distinct fatty acids groups that are based on their chain length, degree of saturation, or traditional chemical groupings used in previous studies. The individual fatty acids in the compositional biplots were plotted very close to each other indicating high correlations among them [26]. Thus, concentrations of fatty acids may not be interpreted independently from each other.

Our results further show that some fatty acids of small constituent sizes are highly variable. The inclusion of these small fatty acids in the analysis of overall composition leads to a shift in balance between fatty acid constituents and to increased variation in other fatty acids of larger constituent size. Similarly to other studies [16,22,27,28], a second analysis and PCA using only 32 fatty acids that had been used in previous research showed fatty acid groups that were not exclusive to n-3 or n-6 fatty acids (Supplemental Figure S2). We acknowledge that there is no standardized list of fatty acids that has to be included in the “sum of total fatty acids”. However, the fewer the fatty acids that are summed, the greater the apparent profile percentage of those reported [29]. Granted that some of the fatty acids of very small constituent size have a putative impact on the other fatty acids of larger constituent sizes, they could have a crucial role in the pathophysiology of the overall fatty acid composition of human milk.

There were no distinct sub-compositional groups of fatty acids from human milk sampled at 6 weeks and 6 months post-delivery. However, the compositional biplots of human milk collected at 6 months showed some changes in the position and direction of the individual fatty acids compared to the 6 weeks biplots. Although these changes were not striking, they only became clearer at 12 months when all fatty acids could be combined into one of four sub-compositional groups based on their correlational properties. This is a clear indication that the fatty acid profile of human milk during prolonged lactation is not identical to that of human milk during short-term lactation. These changes in human milk fatty acid composition could be reflective of the mother’s metabolic adaptation to the changing needs of the growing infant, as well as mammary gland biosynthetic capacity [30]. However, whether these profiles are independent of the frequency, exclusivity, or type of breastfeeding still remains to be explored.

Amongst the several factors shown to influence human milk fatty acid composition, maternal dietary habits have a short- and long-term impact on human milk fatty acid composition [31]. Although we do not have dietary data from the lactating mothers, we used the child indicator food index (assessed at ages four and five years) as a remote proxy for maternal diet. A comparatively simple index was calculated based on the consumption of seven food groups [32] and was used as an indicator of a favorable, neutral, or unfavorable diet. Our results show that more than 50% of the children at ages four and five years, potentially extending to the whole family including the mother, were most likely consuming a favorable diet. In addition, maternal age, higher education, and subsequent lower rates of individual risk factors including obesity were previously found to be closely associated with prolonged breastfeeding [17]. Therefore, granted that the majority of lactating women were highly educated, of high maternal age, and of presumably high socio-economic status, it is plausible that they were taking some supplements, perhaps even fish oil, which could have driven the PCA results. Thus, we attribute the changes in human milk fatty acid profile at 12 months to an increased stability of the mother’s diet as well as other lifestyle changes that are associated with a longer duration of lactation.

We identified a sub-compositional cluster which comprised TFAs from partially hydrogenated vegetable oils (PHVOs; C18:1n-9t), BCFAs (C15:0ai, C16:0i, C18:0i), and the odd chain fatty acids (OCFAs; C15:0, C17:0) found in ruminant fats, from a typical western diet [33]. TFAs are typically used as biomarkers for dietary intake. Thus this compositional cluster remained unchanged during lactation, suggesting that the secretion of TFAs, BCFAs, and OCFAs remains stable during lactation. In addition, TFAs have been associated with adverse effects with respect to essential fatty acid and long-chain PUFA (LCPUFA) metabolism in humans [34], oxidative stress, and infant development [35]. Similar to the previous Ulm birth cohort study [9] and earlier studies [36] in which researchers reported inverse correlations between TFAs and various n-3 and n-6 PUFAs, this fatty acid sub-compositional group comprising TFAs had loadings that are in the opposite direction of the n-3/n-6 dominated sub-compositional group.

Furthermore, a second sub-compositional group was characterized by the essential fatty acid ALA, medium-chain SFAs (C10:0-C14:0), and the long-chain SFA palmitic acid (C16:0) that are acquired through endogenous synthesis and dietary intake [37]. The benefits of individual SFAs in infant nutrition are an eminent topic of interest. Capric acid (C10:0) and lauric acid (C12:0) are linked to antimicrobial biological activities [38], while myristic acid (C14:0) and palmitic exhibit specific properties for protein acylation [39]. It is also noteworthy that ALA is an essential fatty acid that can influence metabolic processes and is important for the endogenous synthesis of its respective LCPUFA. Even though some of the individual SFAs and n-3 PUFAs remained statistically unchanged at 6 and 12 months, this sub-compositional group was significantly higher at 6 months and 12 months of lactation. Additionally, the relative positions and direction of these individual fatty acids changed, suggesting proportional response of the mammary gland as the needs of the infants change during lactation. Thus, the physiological and health consequences associated with these compositional changes are not known and would require further exploration.

Moreover, our results showed a significant decrease of the sub-compositional group which predominantly comprised long-chain n-3 and n-6 fatty acids at 12 months. These results are in contrast to other studies that found marked increases in concentrations of DHA in human milk at 6 months [9] and 9 months [24]. Also, while stearic acid (C18:0) and oleic acid (C18:1n-9) formed part of this sub-compositional group, their importance in infants have not yet been explored in detail in spite of their abundance and regulation within the mammary gland during fat synthesis and lactation. Arachidonic acid (AA, C20:4n-6) and adrenic acid (C22:4n-6) formed a sub-compositional group which was further characterized by long-chain SFAs (C19:0-C24:0) and MUFAs including nervonic acid (C24:1n-9) and docosapentaenoic acid (C22:5n-3). Although this sub-compositional group remained statistically unchanged throughout lactation, MUFAs are the second most common proportion in human milk, but their nutritional relevance and potential functionalities have not been explored in infants [40]. A potentially important aspect about the conversion on AA in vivo is that it functions an immediate precursor of adrenic acid [41]. However, just like AA, rapid accretion of adrenic acid occurs during the early postnatal period on the brain growth spurt in infants. Thus this particular combination of essential and non-essential fatty acids is of strong interest and requires further clinical or population-based investigations to identify and better understand the pathophysiology of these fatty acids. Nonetheless, both mammary gland activity such as de novo lipogenesis and the digestion have a considerable effect on the human milk fatty acid profile.

A limitation of this study is the lack of direct dietary data and potential for selection bias thus the data cannot be used to generalize information regarding all lactating women. Thus, the observational nature of this study restricts the possibility of drawing conclusions on causation, particularly between fatty acid profile, diet and socio-economic status. However, a strength of this study is the human milk samples available at three times points of lactation. Our findings support the hypothesis that a combination of multiple fatty acids is important in fatty acid profiling beyond the presentation of individual fatty acids. Thus, selecting a statistically appropriate balance may help to avoid spurious results and better address the biological mechanisms associated with the changes in human milk fatty acid composition. It is also important to consider the high variability of small fatty acids and this should be awarded attention because a compositional analysis may lead to more pronounced changes in the human milk fatty acid profile. Although exploring human milk fatty acid compositions using a more standard approach like chemical-based groupings could be methodologically constant, this may not be robust enough.

## 5. Conclusions

In conclusion, the fatty acid composition of human milk sampled at 6 weeks, 6 months, and 12 months may not group in similar sub-compositional patterns. Thus, the composition of human milk during prolonged lactation is different from the composition of human milk during short duration of lactation. However, whether these changes are associated with specific improved developmental or health outcomes in infants still remains to be studied. We recommend further clinical or population-based investigations to identify and better understand the pathophysiology of the different sub-compositional fatty acids groups in human milk during lactation.

## Figures and Tables

**Table 1:** Characteristics of the lactating women included in the Ulmer SPATZ Health study.

Characteristic	All 6 Weeks Samples (*n* = 706)		All 6 Months Samples (*n* = 483)		All 12 Months Samples (*n* = 83)	
	*n*	% or Mean	*n*	% or Mean	*n*	% or Mean
Age	706	33.1	483	33.5	83	34.4
Gestation period						
Early (37 or 38 weeks)	146	22.5%	96	21.5%	18	22.8%
Full (39 or 40 weeks)	397	61.3%	275	61.5%	47	59.5%
Late or Post (41 or 42 weeks)	105	16.2%	76	17.0%	14	17.7%
Parity (*n* births of fetus ≥24 weeks)						
0 births	368	52.2%	252	52.2%	39	47.0%
≥1 birth	337	47.8%	231	47.8%	44	53.0%
Delivery mode						
Vaginal spontaneous	480	68.1%	344	71.2%	60	72.3%
Elective caesarean	69	9.8%	39	8.1%	9	10.8%
Emergency caesarean	90	12.8%	56	11.6%	9	10.8%
Vaginal assisted	66	9.4%	44	9.1%	5	6.0%
Maternal pre-pregnancy body mass index (BMI) category						
Underweight (BMI < 18.50)	12	1.8%	6	1.3%	3	3.8%
Normal (18.50 ≤ BMI < 25.00)	443	64.8%	325	69.3%	55	68.8%
Overweight (25.00 ≤ BMI < 30.00)	150	21.9%	93	19.8%	15	18.8%
Obese (BMI > 30.00)	79	11.5%	45	9.6%	7	8.8%
Maternal BMI category (6 weeks)						
Underweight	6	1.0%	3	0.7%		
Normal	364	58.0%	268	60.4%	45	56.3%
Overweight	189	30.1%	131	29.5%	27	33.8%
Obese	69	11.0%	42	9.5%	8	10.0%
Maternal BMI category (6 months)						
Underweight	15	2.6%	10	2.4%	3	3.9%
Normal	360	62.8%	277	66.4%	50	64.9%
Overweight	136	23.7%	92	22.1%	18	23.4%
Obese	62	10.8%	38	9.1%	6	7.8%
Education level						
Low	43	6.2%	16	3.4%	3	3.7%
Intermediate	196	28.2%	115	24.2%	13	16.0%
High	455	65.6%	345	72.5%	65	80.2%
Occupation						
Leadership/professionals	148	30.3%	102	30.2%	20	34.5%
Intermediate position	223	45.6%	160	47.3%	27	46.6%
Skilled manuals/non-manuals	88	18.0%	63	18.6%	8	13.8%
Unskilled/semi-skilled	18	3.7%	5	1.5%	1	1.7%
Self-employed	12	2.5%	8	2.4%	2	3.4%
Indicator food index (child, age 4)						
Neutral	221	44.2%	153	40.7%	28	38.9%
Favorable	279	55.8%	223	59.3%	44	61.1%
Indicator food index (child, age 5)						
Neutral	198	45.3%	150	44.9%	29	43.9%
Favorable	237	54.2%	182	54.5%	37	56.1%
Unfavorable	2	0.5%	2	0.6%		
Maternal alcohol consumption						
Never drinker	165	23.4%	114	23.6%	40	48.2%
Abstinent drinker	203	28.8%	173	35.8%	24	28.9%
Resumed drinking at 6 weeks to 6 months	150	21.2%	90	18.6%	8	9.6%
Resumed drinking by 6 weeks	115	16.3%	73	15.1%	10	12.0%
Undetermined drinking	73	10.3%	33	6.8%	1	1.2%
Smoking relapse status by 2 years						
Non-smoker	539	78.3%	394	82.8%	74	89.2%
Relapse by 6 weeks	48	7.0%	16	3.4%	2	2.4%
Relapse by 6 months	22	3.2%	7	1.5%		
Relapse 6 months to 2 years	27	3.9%	18	3.8%		
Abstained up to 2 years	52	7.6%	41	8.6%	7	8.4%

Sums (*n*) may not always add up to total because of missing data for certain items. Percentages exclude missing data.

**Table 2:** Means and standard deviations of centered log ratio (clr)-transformed fatty acid concentrations of human milk samples measured at 6 weeks, 6 months, and 12 months of lactation (*n* = 81).

Fatty Acid	Common Name	6 Weeks		6 Months		12 Months		*p* Value ^a^	*p* Value ^b^	*p* Value ^c^
C8:0	Caprylic	−0.07	(0.57)	0.06	(0.47)	0.01	(0.47)	0.2109	0.3500	0.7188
C10:0	Capric	2.03	(0.26)	2.09	(0.22)	2.10	(0.25)	0.1135	0.9546	0.1166
C11:0	Undecylic	−2.25	(0.33)	−2.21	(0.34)	−2.28	(0.34)	0.3313	0.1943	0.6914
C12:0	Lauric	3.25	(0.36)	3.42	(0.31)	3.67	(0.29)	0.0021	<.0001	<.0001
C13:0	Tridecylic	−1.61	(0.24)	−1.55	(0.42)	−1.50	(0.59)	0.0125	0.0357	<.0001
C14:0	Myristic	3.50	(0.23)	3.68	(0.20)	3.98	(0.26)	<.0001	<.0001	<.0001
C15:0	Pentadecylic	0.80	(0.25)	0.86	(0.26)	0.82	(0.25)	0.1090	0.4683	0.3561
C16:0	Palmitic	4.88	(0.15)	4.90	(0.14)	4.91	(0.15)	0.6024	0.3884	0.1903
C17:0	Margaric	0.61	(0.17)	0.64	(0.17)	0.65	(0.16)	0.1620	0.6212	0.0366
C18:0	Stearic	3.69	(0.21)	3.72	(0.21)	3.72	(0.18)	0.4243	0.8974	0.3372
C19:0	Nonadecylic acid	−1.84	(0.27)	−1.80	(0.26)	−1.77	(0.21)	0.1869	0.9213	0.1300
C20:0	Arachidic	0.16	(0.21)	0.14	(0.21)	0.16	(0.23)	0.4986	0.6791	0.7771
C22:0	Behenic	−0.68	(0.26)	−0.70	(0.24)	−0.64	(0.34)	0.5421	0.5839	0.9626
C23:0	Tricosylic	−3.93	(1.99)	−3.36	(1.63)	−3.63	(1.94)	0.0489	0.3875	0.2778
C24:0	Lignoceric	−1.16	(0.51)	−1.18	(0.46)	−1.05	(0.47)	0.5388	0.0927	0.2675
C12:1n-1		−2.23	(0.34)	−2.18	(0.35)	−2.20	(0.38)	0.4302	0.8789	0.5050
C14:1n-5	Myristoleic	0.48	(0.28)	0.53	(0.33)	0.45	(0.33)	0.3022	0.1776	0.7063
C16:1n-7	Palmitoleic	2.55	(0.32)	2.50	(0.27)	2.46	(0.26)	0.2975	0.2921	0.0640
C18:1n-7	Vaccenic	2.19	(0.24)	2.15	(0.19)	2.14	(0.21)	0.0966	0.8250	0.0842
C18:1n-9	Oleic	5.26	(0.20)	5.29	(0.16)	5.25	(0.19)	0.4828	0.1189	0.4663
C20:1n-9	Eicosenoic	0.86	(0.24)	0.76	(0.23)	0.80	(0.26)	0.0035	0.3658	0.0366
C22:1n-9	Erucic	−0.89	(0.29)	−1.02	(0.29)	−0.90	(0.32)	0.0057	0.0133	0.6572
C24:1n-9	Nervonic	−1.62	(0.95)	−1.60	(0.57)	−1.38	(0.56)	0.2704	0.0202	0.3101
C15:0 anteiso	Anteisopentadecylic	−0.44	(0.37)	−0.33	(0.40)	−0.38	(0.45)	0.0221	0.5656	0.0845
C16:0 iso	Isopalmitic	−0.72	(0.31)	−0.64	(0.33)	−0.69	(0.34)	0.0728	0.3149	0.3330
C18:0 iso		−1.89	(0.24)	−1.86	(0.23)	−1.84	(0.24)	0.1615	0.8329	0.1142
C14:1n-5t	Myristelaidic	−4.26	(0.56)	−4.20	(0.51)	−4.25	(0.67)	0.3483	0.3893	0.9213
C15:1n-5t		−4.23	(0.71)	−4.14	(0.48)	−4.19	(0.74)	0.5931	0.8921	0.5851
C16:1n-7t		−1.58	(0.34)	−1.58	(0.35)	−1.60	(0.38)	0.6177	0.9027	0.7263
C18:1n-9t	Elaidic	0.89	(0.54)	1.01	(0.62)	1.04	(0.54)	0.0966	0.9439	0.0386
C18:2n-6tt	Linolelaidic	−0.90	(0.67)	−0.73	(0.50)	−0.97	(0.88)	0.0155	0.0510	0.5908
C18:2n-6	Linoleic	4.03	(0.30)	4.08	(0.33)	4.06	(0.33)	0.4612	0.6236	0.8525
C18:3n-6	γ-linolenic	−0.42	(0.39)	-0.61	(0.37)	−0.93	(0.44)	0.0029	<.0001	<.0001
C20:2n-6	Eicosadienoic	0.36	(0.26)	0.19	(0.28)	0.24	(0.29)	<.0001	0.1177	0.0017
C20:3n-6	Dihomo-γ-linolenic	0.66	(0.27)	0.38	(0.26)	0.31	(0.29)	<.0001	0.1131	<.0001
C20:4n-6	Arachidonic	0.80	(0.27)	0.75	(0.24)	0.79	(0.28)	0.1954	0.3847	0.6236
C22:2n-6	Docosadienoic	−2.13	(0.95)	−2.50	(1.12)	−2.30	(0.93)	<.0001	0.0667	0.0149
C22:4n-6	Adrenic	−0.97	(0.65)	−0.99	(0.29)	−0.85	(0.41)	0.0857	0.0014	0.1321
C22:5n-6	Osbond	−1.62	(0.54)	−1.71	(0.35)	−1.74	(0.48)	0.0122	0.4954	0.0464
C18:3n-3	α -linoleic	1.59	(0.40)	1.67	(0.42)	1.56	(0.36)	0.2960	0.1237	0.7630
C20:3n-3	Dihomo-α-linoleic	−1.54	(0.49)	−1.71	(0.33)	−1.65	(0.32)	0.0002	0.3684	0.0038
C20:4n-3		−0.74	(0.37)	−1.10	(0.35)	−1.32	(0.43)	<.0001	0.0008	<.0001
C20:5n-3	Eicosapentaenoic	−1.01	(0.35)	−1.03	(0.43)	−1.19	(0.39)	0.6142	0.0194	0.0021
C22:5n-3	Docosapentanoic	−0.17	(0.23)	−0.16	(0.21)	−0.06	(0.20)	0.7887	0.0030	0.0027
C22:6n-3	Docosahexaenoic	0.30	(0.44)	0.12	(0.52)	0.20	(0.43)	0.0073	0.2025	0.1004

*p* Values derived from Wilcoxon signed-rank test comparing fatty acid concentrations measured at ^a^ 6 weeks and 6 months, ^b^ 6 months and 12 months, and ^c^ 6 weeks and 12 months. Bonferroni-adjusted level of statistical significance is α = 0.05/45 = 0.0011.

**Table 3:** Changes in human milk fatty acid composition during the first year of lactation.

Fatty Acid	6 Weeks (*n* = 81)		6 Months (*n* = 81)		12 Months (*n* = 81)	
	LS Means (95% CL) ^a^		Estimate	*p* Value	Estimate	*p* Value
**12 Months Derived**						
Group 1	1.4	(1.4, 1.4)	0.037	0.4470	−0.011	0.8028
Group 2	−1.5	(−1.6, −1.5)	0.049	0.0062	0.122	<0.0001
Group 3	1.8	(1.8, 1.9)	−0.007	0.7908	−0.067	0.0029
Group 4	−1.7	(−1.8, −1.7)	−0.079	0.0073	−0.044	0.0998
SFA	2.3	(2.4, 2.4)	0.038	0.1476	0.105	<0.0001
MUFA	2.3	(2.2, 2.3)	−0.021	0.4191	−0.080	0.0012
Trans-FA	−2	(−2.1, −1.8)	0.073	0.4245	0.090	0.2813
BCFAs	−2.9	(−3.0, −2.8)	0.041	0.5426	−0.039	0.5039
PUFA	1.2	(1.1, 1.2)	0.017	0.6632	−0.034	0.2977
∑n-3 PUFA	−1	(−1.1, −0.9)	−0.035	0.4612	−0.111	0.0125
∑n-3 LCPUFA	−1.9	(−2.0, −1.8)	−0.158	0.0003	−0.160	<0.0001
∑n-6 PUFA	1	(1.0, 1.1)	0.024	0.5694	−0.022	0.5128
∑n-6 LCPUFA	−1.2	(−1.3, −1.2)	−0.178	<.0001	−0.171	<0.0001

^a^ Least Square Means (95% Confidence Limits). 12 months: Group 1 (C18:0i, C16:0i, C15:0ai, C16:1n-7t, C18:1n-9t, C18:2n-6tt, C14:1n-5t, C12:1n-1, C14:1n-5, C15:0, C17:0); Group 2 (C10:0, C11:0, C12:0, C13:0, C14:0, C16:0, C15:1n-5t, C18:3n-3); Group 3 (C20:5n-3, C22:6n-3, C20:4n-3, C20:3n-6, C8:0, C18:3n-6, C18:2n-6, C18:0, C22:5n-6, C18:1n-7, C22:2n-6, C20:3n-6, C20:2n-6, C18:1n-9); Group 4 (C19:0, C20:0, C22:0, C23:0, C24:0, C20:1n-9, C24:1n-9, C22:1n-9, C22:5n-3, C22:4n-6, C20:4n-6). SFA: saturated fatty acid; MUFA: monounsaturated fatty acid; Trans-FA: trans-fatty acid; BCFA: branched chain fatty acid; PUFA: polyunsaturated fatty acid; LCPUFA: Long-chain polyunsaturated fatty acid; Maternal age, education, occupation, parity, pre-pregnancy BMI, and delivery mode were added as covariates. Bonferroni adjusted level of statistical significance: α = 0.05/4 = 0.0125.

## Supplementary Materials

The following are available online at https://www.mdpi.com/2072-6643/11/12/2842/s1, Table S1: Means and standard deviations for clr-transformed fatty acid concentrations from all human milk samples measured at 6 weeks, 6 months, and 12 months of lactation, Figure S1: Compositional biplots from principal component analysis (PCA) of centered log-ratio transformed fatty acid concentrations of human milk samples collected at 6 weeks and 6 months, Table S2: Changes in human milk fatty acid composition during the first 6 months of lactation, Table S3: Means and standard deviation for selected (32) clr-transformed individual fatty acids from human milk samples collected at 6 weeks, 6 months, and 12 months post-delivery, Figure S2: Compositional biplots from principal component analysis (PCA) of selected (32) clr-transformed fatty acid from human milk samples collected at 6 weeks, 6 months, and 12 months post-delivery, Table S3: Means and standard deviation for individual fatty acids from human milk samples collected at 6 weeks, 6 months, and 12 months post-delivery (%weight of total fatty acids).

## References

[1] Ballard O., Morrow A.L. (2013). Human milk composition: Nutrients and bioactive factors. Pediatric Clin..

[2] Grote V., Verduci E., Vecchi F., Contarini G., Giovannini M., Koletzko B. (2016). Breast milk composition and infant nutrient intakes during the first 12 months of life. Eur. J. Clin. Nutr..

[3] Koletzko B., Bhutta Z.A., Cai W., Cruchetm S., Guindim M.E., Fuchs G.J. (2013). Compositional Requirements of Follow-Up Formula for Use in Infancy: Recommendations of an International Expert Group Coordinated by the Early Nutrition Academy. Ann. Nutr. Metab..

[4] Innis S.M. (2014). Impact of maternal diet on human milk composition and neurological development of infants. Am. J. Clin. Nutr..

[5] Roy S., Dhar P., Ghosh S. (2012). Comparative evaluation of essential fatty acid composition of mothers’ milk of some urban and suburban regions of West Bengal, India. Int. J. Food Sci. Nutr..

[6] Olafsdottir A.S., Thorsdottir I., Wagner K.H., Elmadfa I. (2006). Polyunsaturated fatty acids in the diet and breast milk of lactating icelandic women with traditional fish and cod liver oil consumption. Ann. Nutr. Metab..

[7] Fu Y., Liu X., Zhou B., Jiang A.C., Chai L. (2016). An updated review of worldwide levels of docosahexaenoic and arachidonic acid in human breast milk by region. Public Health Nutr..

[8] Much D., Brunner S., Vollhardt C., Schmid D., Sedlmeier E.-M., Brüderl M. (2013). Breast milk fatty acid profile in relation to infant growth and body composition: Results from the INFAT study. Pediatric Res..

[9] Szabo E., Boehm G., Beermann C., Weyermann M., Brenner H., Rothenbacher D. (2010). Fatty acid profile comparisons in human milk sampled from the same mothers at the sixth week and the sixth month of lactation. J. Pediatric Gastroenterol. Nutr..

[10] Fidler N., Sauerwald T., Pohl A., Demmelmair H., Koletzko B. (2000). Docosahexaenoic acid transfer into human milk after dietary supplementation: A randomized clinical trial. J. Lipid Res..

[11] Agostoni C., Marangoni F., Lammardo A.M., Galli C., Giovannini M., Riva E. (2001). Long-chain polyunsaturated fatty acid concentrations in human hindmilk are constant throughout twelve months of lactation. Adv. Exp. Med. Biol..

[12] Marangoni F., Agostoni C., Lammardo A.M., Giovannini M., Galli C., Riva E. (2000). Polyunsaturated fatty acid concentrations in human hindmilk are stable throughout 12-months of lactation and provide a sustained intake to the infant during exclusive breastfeeding: An Italian study. Br. J. Nutr..

[13] Barreiro R., Regal P., López-Racamonde O., Cepeda A., Fente C.A. (2018). Comparison of the fatty acid profile of Spanish infant formulas and Galician women breast milk. J. Physiol. Biochem..

[14] Aitchison J.J., Egozcue J. (2005). Compositional Data Analysis: Where Are We and Where Should We Be Heading?. Math. Geol..

[15] Hodson L., Skeaff C.M., Fielding B.A. (2008). Fatty acid composition of adipose tissue and blood in humans and its use as a biomarker of dietary intake. Prog. Lipid Res..

[16] Voortman T., Tielemans M.J., Stroobant W., Schoufour J.D., Kiefte-de Jong J.C., Steenweg-de Graaff J. (2018). Plasma fatty acid patterns during pregnancy and child’s growth, body composition, and cardiometabolic health: The Generation R Study. Clin. Nutr..

[17] Logan C., Zittel T., Striebel S., Reister F., Brenner H., Rothenbacher D. (2016). Changing Societal and Lifestyle Factors and Breastfeeding Patterns Over Time. Pediatrics.

[18] Bligh E.G., Dyer W.J. (1959). A rapid method of total lipid extraction and purification. Can. J. Biochem. Physiol..

[19] Beermann C., Green A., Möbius M., Schmitt J.J., Boehm G. (2003). Lipid class separation by HPLC combined with GC FA analysis: Comparison of seed lipid compositions from different Brassica napus L. varieties. J. Am. Oil Chem. Soc..

[20] Lucas A., Gibbs J.A., Lyster R.L., Baum J.D. (1978). Creamatocrit: Simple clinical technique for estimating fat concentration and energy value of human milk. Br. Med. J..

[21] Jones E., Dimmock P.W., Spencer S.A. (2001). A randomised controlled trial to compare methods of milk expression after preterm delivery. Arch. Dis. Child.-Fetal Neonatal Ed..

[22] Logan C.A., Brandt S., Wabitsch M., Brenner H., Wiens F., Stahl B. (2017). New approach shows no association between maternal milk fatty acid composition and childhood wheeze or asthma. Allergy.

[23] Da Cunha J., Macedo da Costa T.H., Ito M.K. (2005). Influences of maternal dietary intake and suckling on breast milk lipid and fatty acid composition in low-income women from Brasilia, Brazil. Early Hum. Dev..

[24] Mandel D., Lubetzky R., Dollberg S., Barak S., Mimouni F.B. (2005). Fat and Energy Contents of Expressed Human Breast Milk in Prolonged Lactation. Pediatrics.

[25] Keating E.M., Curtis B.A., Slusher T.M. (2013). Maternal milk volume and breast milk expression: Implications for diet and nutrition in infants. Handbook of Dietary and Nutritional Aspects of Human Breast Milk.

[26] Ros-Freixedes R., Estany J. (2014). On the Compositional Analysis of Fatty Acids in Pork. J. Agric. Biol. Environ. Stat..

[27] Imamura F., Sharp S.J., Koulman A., Schulze M.B., Kröger J., Griffin J.L. (2017). A combination of plasma phospholipid fatty acids and its association with incidence of type 2 diabetes: The EPIC-InterAct case-cohort study. PLoS Med..

[28] Siziba L.P., Baumgartner J., Ricci C., Jacobs A., Rothman M., Matsungo T.M. (2017). Associations of plasma total phospholipid fatty acid patterns with feeding practices, growth, and psychomotor development in 6-month-old South African infants. Matern. Child Nutr..

[29] Brenna J.T., Plourde M., Stark K.D., Jones P.J., Lin Y.-H. (2018). Best practices for the design, laboratory analysis, and reporting of trials involving fatty acids. Am. J. Clin. Nutr..

[30] Butts C.A., Hedderley D.I., Herath T.D., Paturi G., Glyn-Jones S., Wiens F. (2018). Human Milk Composition and Dietary Intakes of Breastfeeding Women of Different Ethnicity from the Manawatu-Wanganui Region of New Zealand. Nutrients.

[31] Antonakou A., Skenderi K.P., Chiou A., Anastasiou C.A., Bakoula C., Matalas A.L. (2013). Breast milk fat concentration and fatty acid pattern during the first six months in exclusively breastfeeding Greek women. Eur. J. Nutr..

[32] Truthmann J., Richter A., Thiele S., Drescher L., Roosen J., Mensink G.B. (2012). Associations of dietary indices with biomarkers of dietary exposure and cardiovascular status among adolescents in Germany. Nutr. Metab..

[33] Jenkins B., West J.A., Koulman A. (2015). A Review of Odd-Chain Fatty Acid Metabolism and the Role of Pentadecanoic Acid (C15:0) and Heptadecanoic Acid (C17:0) in Health and Disease. Molecules.

[34] Mueller A., Thijs C., Rist L., Simões-Wüst A.P., Huber M., Steinhart H. (2010). Trans Fatty Acids in Human Milk are an Indicator of Different Maternal Dietary Sources Containing Trans Fatty Acids. Lipids.

[35] Visentainer J.V., Santos O.O., Maldaner L., Zappielo C., Neia V.L. (2018). Lipids and Fatty Acids in Human Milk: Benefits and Analysis. Biochem. Health Benefits Fat. Acids.

[36] Innis S.M., King D.J. (1999). trans Fatty acids in human milk are inversely associated with concentrations of essential all-cis n−6 and n−3 fatty acids and determine trans, but not n−6 and n−3, fatty acids in plasma lipids of breast-fed infants. Am. J. Clin. Nutr..

[37] Hudgins L.C., Hellerstein M., Seidman C., Neese R.J., Hirsch J. (1996). Human fatty acid synthesis is stimulated by a eucaloric low fat, high carbohydrate diet. J. Clin. Investig..

[38] Gardner A.S., Rahman I.A., Lai C.T., Hepworth A., Trengove N., Hartmann P.E. (2017). Changes in Fatty Acid Composition of Human Milk in Response to Cold-Like Symptoms in the Lactating Mother and Infant. Nutrients.

[39] Høstmark A.T., Haug A. (2013). Percentages of oleic acid and arachidonic acid are inversely related in phospholipids of human sera. Lipids Health Dis..

[40] Delplanque B., Gibson R., Koletzko B., Lapillonne A., Strandvik B. (2015). Lipid Quality in Infant Nutrition: Current Knowledge and Future Opportunities. J. Pediatric Gastroenterol. Nutr..

[41] Hadley K., Ryan A., Forsyth S., Gautier S., Salem N. (2016). The Essentiality of Arachidonic Acid in Infant Development. Nutrients.

