# Supplementary material for: Changes in Human Milk Fatty Acid Composition during Lactation: The Ulm SPATZ Health Study
Source: Nutrients. 2019 Nov 20;11(12):2842. doi: 10.3390/nu11122842 (PMC6949892; doi:10.3390/nu11122842)
Supplement: Supplementary file 1 [file nutrients-11-02842-s001.zip › Supplementary Figures and Tables_Revised.docx]

**SUPPLEMENTAL MATERIAL**

Table S1: Means and standard deviations for clr-transformed fatty acid concentrations from all human milk samples measured at 6 weeks, 6 months and 12 months of lactation

| **Fatty Acid** | **Common name** | **6 weeks**  **(n=706)** | | **6 months**  **(n=483)** | | **12 months**  **(n=83)** | | **Pvalue^1^** |
| --- | --- | --- | --- | --- | --- | --- | --- | --- |
| C8:0 | Caprylic | 0.24 | (0.47) | 0.28 | (0.36) | 0.01 | (0.47) | 0.0033 |
| C10:0 | Capric | 2.29 | (0.30) | 2.33 | (0.28) | 2.10 | (0.25) | <.0001 |
| C11:0 | Undecylic | -2.31 | (0.30) | -2.27 | (0.29) | -2.28 | (0.34) | 0.0163 |
| C12:0 | Lauric | 3.45 | (0.34) | 3.62 | (0.31) | 3.67 | (0.29) | <.0001 |
| C13:0 | Tridecylic | -1.57 | (0.24) | -1.47 | (0.28) | -1.50 | (0.59) | <.0001 |
| C14:0 | Myristic | 3.57 | (0.23) | 3.78 | (0.23) | 3.98 | (0.26) | <.0001 |
| C15:0 | Pentadecylic | 0.79 | (0.24) | 0.86 | (0.23) | 0.82 | (0.25) | 0.0002 |
| C16:0 | Palmitic | 4.88 | (0.14) | 4.92 | (0.14) | 4.91 | (0.15) | <.0001 |
| C17:0 | Margaric | 0.59 | (0.16) | 0.66 | (0.16) | 0.65 | (0.16) | <.0001 |
| C18:0 | Stearic | 3.68 | (0.22) | 3.76 | (0.22) | 3.72 | (0.18) | <.0001 |
| C19:0 | Nonadecylic acid | -1.85 | (0.26) | -1.79 | (0.35) | -1.77 | (0.21) | <.0001 |
| C20:0 | Arachidic | 0.16 | (0.23) | 0.20 | (0.24) | 0.16 | (0.23) | 0.0026 |
| C22:0 | Behenic | -0.70 | (0.24) | -0.66 | (0.27) | -0.64 | (0.34) | 0.0009 |
| C23:0 | Tricosylic | -4.37 | (2.13) | -4.07 | (2.07) | -3.63 | (1.94) | 0.0003 |
| C24:0 | Lignoceric | -1.10 | (0.38) | -1.20 | (0.44) | -1.05 | (0.47) | 0.0359 |
| C12:1n-1 |  | -2.11 | (0.37) | -2.08 | (0.37) | -2.20 | (0.38) | 0.1332 |
| C14:1n-5 | Myristoleic | 0.52 | (0.28) | 0.57 | (0.28) | 0.45 | (0.33) | 0.0738 |
| C16:1n-7 | Palmitoleic | 2.63 | (0.30) | 2.60 | (0.28) | 2.46 | (0.26) | 0.0667 |
| C18:1n-7 | Vaccenic | 2.27 | (0.24) | 2.24 | (0.25) | 2.14 | (0.21) | 0.0442 |
| C18:1n-9 | Oleic | 5.30 | (0.19) | 5.34 | (0.18) | 5.25 | (0.19) | 0.0001 |
| C20:1n-9 | Eicosenoic | 0.91 | (0.22) | 0.84 | (0.23) | 0.80 | (0.26) | <.0001 |
| C22:1n-9 | Erucic | -0.81 | (0.26) | -0.92 | (0.28) | -0.90 | (0.32) | <.0001 |
| C24:1n-9 | Nervonic | -1.11 | (0.61) | -1.25 | (0.52) | -1.38 | (0.56) | <.0001 |
| C15:0 anteiso | Anteisopentadecylic | -0.45 | (0.39) | -0.34 | (0.35) | -0.38 | (0.45) | <.0001 |
| C16:0 iso | Isopalmitic | -0.73 | (0.30) | -0.64 | (0.33) | -0.69 | (0.34) | <.0001 |
| C18:0i |  | -1.90 | (0.27) | -1.84 | (0.34) | -1.84 | (0.24) | <.0001 |
| C14:1n-5t | Myristelaidic | -4.25 | (0.59) | -4.20 | (0.56) | -4.25 | (0.67) | 0.2586 |
| C15:1n-5t |  | -4.18 | (0.54) | -4.15 | (0.50) | -4.19 | (0.74) | 0.6865 |
| C16:1n-7t |  | -2.03 | (0.56) | -1.98 | (0.57) | -1.60 | (0.38) | 0.7161 |
| C18:1n-9t | Elaidic | 0.73 | (0.80) | 0.83 | (0.85) | 1.04 | (0.54) | 0.0225 |
| C18:2n-6tt | Linolelaidic | -2.58 | (1.46) | -2.25 | (1.50) | -0.97 | (0.88) | 0.0424 |
| C18:2n-6 | Linoleic | 4.05 | (0.31) | 4.09 | (0.28) | 4.06 | (0.33) | 0.0011 |
| C18:3n-6 | γ-linolenic | -0.43 | (0.36) | -0.56 | (0.35) | -0.93 | (0.44) | <.0001 |
| C20:2n-6 | Eicosadienoic | 0.43 | (0.25) | 0.25 | (0.24) | 0.24 | (0.29) | <.0001 |
| C20:3n-6 | Dihomo-γ-linolenic | 0.69 | (0.25) | 0.41 | (0.24) | 0.31 | (0.29) | <.0001 |
| C20:4n-6 | Arachidonic | 0.88 | (0.22) | 0.82 | (0.22) | 0.79 | (0.28) | 0.0068 |
| C22:2n-6 | Docosadienoic | -1.90 | (0.66) | -2.31 | (1.00) | -2.30 | (0.93) | <.0001 |
| C22:4n-6 | Adrenic | -0.75 | (0.37) | -0.84 | (0.34) | -0.85 | (0.41) | 0.0040 |
| C22:5n-6 | Osbond | -1.51 | (0.37) | -1.67 | (0.38) | -1.74 | (0.48) | <.0001 |
| C18:3n-3 | α -linoleic | 1.60 | (0.39) | 1.70 | (0.38) | 1.56 | (0.36) | 0.0004 |
| C20:3n-3 | Dihomo-α-linoleic | -1.42 | (0.40) | -1.58 | (0.47) | -1.65 | (0.32) | <.0001 |
| C20:4n-3 |  | -0.72 | (0.32) | -1.05 | (0.32) | -1.32 | (0.43) | <.0001 |
| C20:5n-3 | Eicosapentaenoic | -1.01 | (0.37) | -1.01 | (0.39) | -1.19 | (0.39) | 0.0908 |
| C22:5n-3 | Docosapentanoic | -0.20 | (0.22) | -0.15 | (0.22) | -0.06 | (0.20) | 0.0025 |
| C22:6n-3 | Docosahexaenoic | 0.34 | (0.38) | 0.20 | (0.43) | 0.20 | (0.43) | <.0001 |

P values derived from Wilcoxon signed-rank test comparing fatty acid concentrations measured at 16 weeks restricted to those measured at 6 months (n=467). Bonferroni-adjusted level of statistical significance is α=0.05/45 = 0.0011.


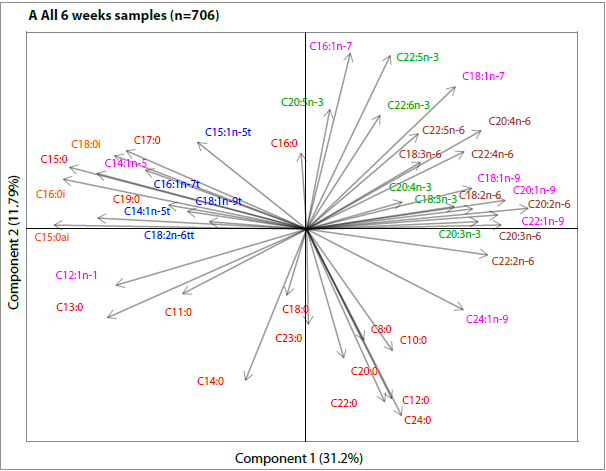

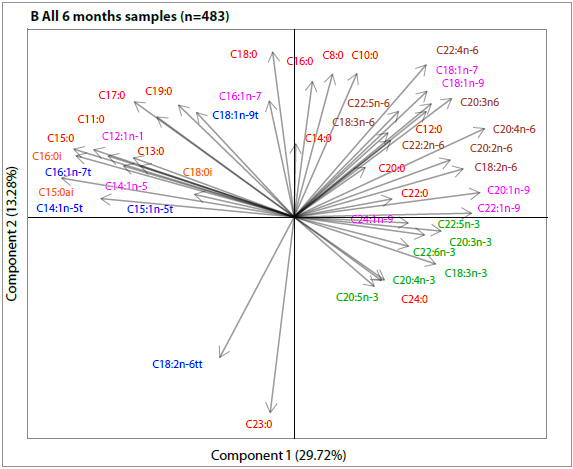

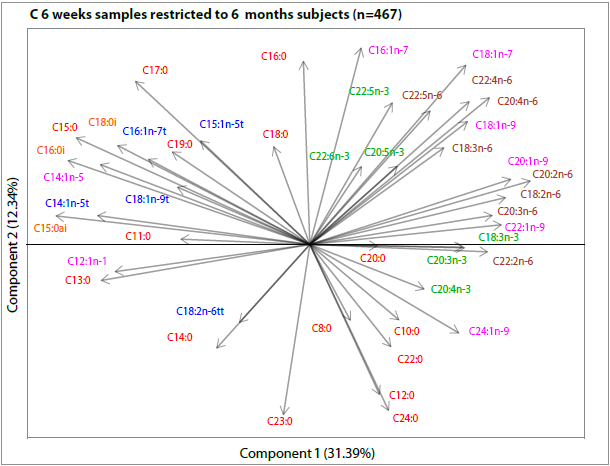

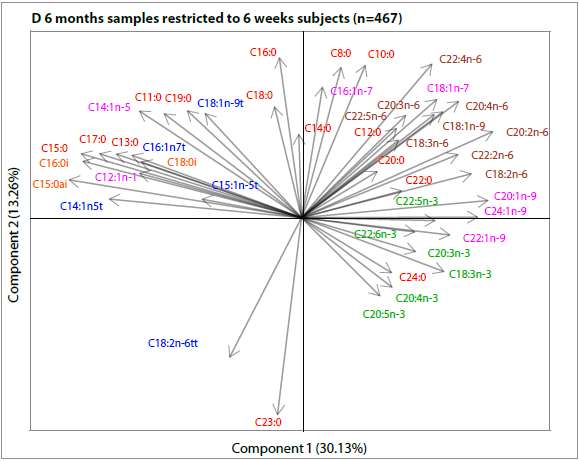

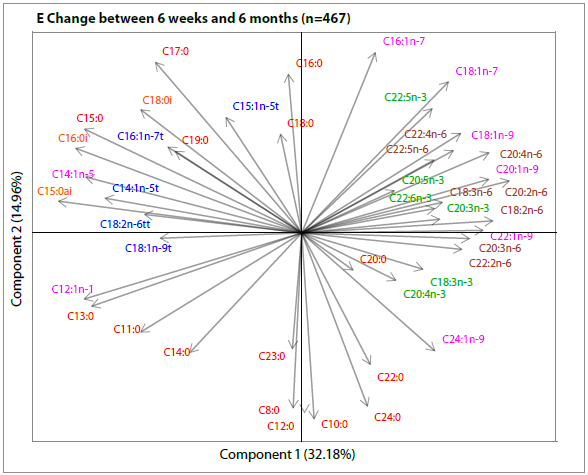


Colour key: Saturated; Trans-; Omega-3; Omega-6; Monounsaturated; Branched-chain

Figure S1: Compositional biplots from principal component analysis (PCA) of centered log-ratio transformed fatty acid concentrations of human milk samples collected at 6 weeks and 6 months

Table S2: Changes in human milk fatty acid composition during the first 6 months of lactation

| **Fatty acid** | **Common name** | **6 weeks (n=467)** | | **6 months (n=467)** | |
| --- | --- | --- | --- | --- | --- |
|  |  | **LS Means (95% CL)** | | **Estimate** | **p value** |
| SFAs |  | 2.4 | (2.4, 2.4) | 0.071 | <.0001 |
| C12:0 | Lauric | 3.4 | (3.4, 3.4) | 0.227 | <.0001 |
| C13:0 | Tridecylic | -1.6 | (-1.6, -1.6) | 0.108 | <.0001 |
| C14:0 | Myristic | 3.5 | (3.5, 3.6) | 0.231 | <.0001 |
| C15:0 | Pentadecylic | 0.8 | (0.8, 0.8) | 0.055 | <.0001 |
| C16:0 | Palmitic | 4.9 | (4.9, 4.9) | 0.044 | <.0001 |
| C17:0 | Margaric | 0.6 | (0.6, 0.6) | 0.062 | <.0001 |
| C18:0 | Stearic | 3.7 | (3.7, 3.8) | 0.079 | <.0001 |
| C19:0 | Nonadecylic acid | -1.9 | (-1.9, -1.8) | 0.054 | 0.0006 |
| C22:0 | Behenic | -0.7 | (-0.7, -0.7) | 0.052 | 0.0002 |
| C23:0 | Tricosylic | -4.3 | (-4.5, -4.1) | 0.205 | 0.0032 |
| MUFAs |  | 2,3 | (2.3, 2.3) | 0.008 | 0.2567 |
| C18:1n-9 | Oleic | 5.3 | (5.3, 5.3) | 0.047 | <.0001 |
| C20:1n-9 | Eicosenoic | 0.9 | (0.9, 0.9) | -0.061 | <.0001 |
| C22:1n-9 | Erucic | -0.8 | (-0.9, -0.8) | -0.094 | <.0001 |
| C24:1n-9 | Nervonic | -1.2 | (-1.2, -1.1) | -0.095 | <.0001 |
| Trans-FAs |  | -2,2 | (-2.2, -2.1) | 0.071 | 0.0155 |
| BCFAs |  | -2.9 | (-2.9, -2.8) | 0.047 | 0.0079 |
| C15:0ai | Anteisopentadecylic | -0.4 | (-0.5, -0.4) | 0.083 | <.0001 |
| C16:0i | Isopalmitic | -0.7 | (-0.7, -0.7) | 0.063 | <.0001 |
| C18:0i |  | -1.9 | (-1.9, -1.9) | 0.065 | <.0001 |
| PUFAs |  | 1.2 | (1.1, 1.2) | 0.011 | 0.3018 |
| C18:3n-3 | α-linoleic | 1.6 | (1.6, 1.6) | 0.091 | <.0001 |
| C20:3n-3 | Dihomo-α-linoleic | -1.4 | (-1.5, -1.4) | -0.161 | <.0001 |
| C20:4n-3 |  | -0.7 | (-0.7, -0.7) | -0.344 | <.0001 |
| C22:6n-3 | Docosahexaenoic | 0.3 | (0.3, 0.4) | -0.141 | <.0001 |
| ∑n-3 PUFA |  | -1,0 | (-1.0, -1.0) | -0.013 | 0.3398 |
| ∑n-3 LCPUFA |  | -1,9 | (-1.9, -1.9) | -0.134 | <.0001 |
| C18:2n-6 | Linoleic | 4.0 | (4.0, 4.1) | 0.065 | <.0001 |
| C18:3n-6 | γ-linolenic | -0.4 | (-0.4, -0.4) | -0.143 | <.0001 |
| C20:2n-6 | Eicosadienoic | 0.4 | (0.4, 0.4) | -0.144 | <.0001 |
| C20:3n-6 | Dihomo-γ-linolenic | 0.7 | (0.7, 0.7) | -0.267 | <.0001 |
| C22:2n-6 | Docosadienoic | -2.0 | (-2.0, -1.9) | -0.362 | <.0001 |
| C22:5n-6 | Osbond | -1.5 | (-1.6, -1.5) | -0.134 | <.0001 |
| ∑n-6 PUFA |  | 1.0 | (1.0, 1.1) | 0.015 | 0.2087 |
| ∑n-6 LCPUFA |  | -1.2 | (-1.2, -1.2) | -0.162 | <.0001 |

SFAs – Saturated Fatty Acids; MUFAs- Monounsaturated Fatty Acids; FAs- Fatty Acids; BCFAs- Branched Chain Fatty Acids; PUFA- Polyunsaturated Fatty Acids; LCPUFA- Long-chain Polyunsaturated Fatty Acids.

Maternal age, parity, pre-pregnancy BMI, and delivery mode were added as covariates.

Bonferroni adjusted level of significance is α=0.05/45=0.0014.

Table S3: Means and standard deviation for selected (32) clr-transformed individual fatty acids from human milk samples collected at 6 weeks, 6 months and 12 months post-delivery

| **Fatty Acid** | | **6 weeks** | | **6 months** | | **12 months** | | **P value^1^** | **P value^2^** | **P value^3^** |
| --- | --- | --- | --- | --- | --- | --- | --- | --- | --- | --- |
| C10:0 | Capric | 1.06 | (0.27) | 1.12 | (0.25) | 0.93 | (0.25) | <.0001 | 0.771 | 0.017 |
| C12:0 | Lauric | 2.22 | (0.32) | 2.41 | (0.28) | 2.50 | (0.26) | <.0001 | <.0001 | <.0001 |
| C14:0 | Myristate | 2.34 | (0.25) | 2.57 | (0.24) | 2.81 | (0.24) | <.0001 | <.0001 | <.0001 |
| C15:0 | Pentadecylic | -0.44 | (0.30) | -0.34 | (0.29) | -0.34 | (0.33) | <.0001 | 0.376 | 0.166 |
| C16:0 | Palmitate | 3.65 | (0.14) | 3.72 | (0.14) | 3.75 | (0.14) | <.0001 | 0.890 | 0.039 |
| C17:0 | Margaric | -0.64 | (0.21) | -0.55 | (0.19) | -0.52 | (0.22) | <.0001 | 0.904 | 0.021 |
| C18:0 | Stearate | 2.45 | (0.23) | 2.55 | (0.22) | 2.56 | (0.20) | <.0001 | 0.607 | 0.139 |
| C20:0 | Arachidic | -1.07 | (0.23) | -1.01 | (0.23) | -1.00 | (0.22) | <.0001 | 0.984 | 0.626 |
| C22:0 | Behenic | -1.93 | (0.24) | -1.87 | (0.26) | -1.81 | (0.32) | <.0001 | 0.710 | 0.514 |
| C24:0 | Lignoceric | -2.33 | (0.37) | -2.41 | (0.45) | -2.22 | (0.47) | 0.145 | 0.141 | 0.196 |
| C16:1n7t |  | -3.25 | (0.55) | -3.18 | (0.59) | -2.77 | (0.45) | 0.965 | 0.691 | 0.468 |
| C18:1n9t | Elaidic | -0.44 | (0.56) | -0.31 | (0.61) | -0.12 | (0.59) | 0.007 | 0.834 | 0.030 |
| C14:1n5 | Myristoleic | -0.71 | (0.33) | -0.64 | (0.33) | -0.72 | (0.40) | 0.006 | 0.127 | 0.836 |
| C16:1n7 | Palmitoleate | 1.40 | (0.28) | 1.39 | (0.27) | 1.30 | (0.26) | 0.362 | 0.171 | 0.110 |
| C18:1n7 | Vaccenate | 1.04 | (0.20) | 1.03 | (0.22) | 0.98 | (0.15) | 0.631 | 0.271 | 0.160 |
| C18:1n9 | Oleic | 4.07 | (0.14) | 4.14 | (0.13) | 4.09 | (0.14) | <.0001 | 0.012 | 0.711 |
| C20:1n9 | Eicosenoic | -0.32 | (0.17) | -0.37 | (0.19) | -0.37 | (0.20) | <.0001 | 0.814 | 0.052 |
| C24:1n9 | Nervonic | -2.32 | (0.51) | -2.44 | (0.44) | -2.55 | (0.52) | <.0001 | 0.016 | 0.263 |
| C22:1n9 | Erucic | -2.04 | (0.22) | -2.13 | (0.24) | -2.07 | (0.27) | <.0001 | 0.023 | 0.892 |
| C18:2n6 | Linoleic | 2.82 | (0.27) | 2.89 | (0.24) | 2.90 | (0.26) | <.0001 | 0.382 | 0.323 |
| C18:3n6 | γ-linolenic | -1.66 | (0.33) | -1.77 | (0.32) | -2.09 | (0.40) | <.0001 | <.0001 | <.0001 |
| C20:2n6 | Docosadienoic | -0.81 | (0.20) | -0.95 | (0.19) | -0.92 | (0.21) | <.0001 | 0.162 | 0.001 |
| C20:3n6 | Dihomo-γ-linolenic | -0.54 | (0.21) | -0.80 | (0.20) | -0.85 | (0.22) | <.0001 | 0.021 | <.0001 |
| C20:4n6 | Arachidonic | -0.35 | (0.16) | -0.38 | 0.17) | -0.38 | (0.20) | 0.025 | 0.571 | 0.973 |
| C22:2n6 | Docosadienoic | -3.08 | (0.33) | -3.31 | (0.42) | -3.33 | (0.39) | <.0001 | 0.159 | 0.007 |
| C22:4n6 | Adrenic | -1.98 | (0.29) | -2.04 | (0.30) | -2.01 | (0.37) | 0.020 | <0.001 | 0.014 |
| C22:5n6 | Osbond | -2.73 | (0.27) | -2.86 | (0.27) | -2.86 | (0.27) | <.0001 | 0.760 | 0.045 |
| C18:3n3 | α-linoleic | 0.37 | (0.36) | 0.49 | (0.36) | 0.39 | (0.32) | <.0001 | 0.054 | 0.973 |
| C20:3n3 | Eicosatrienoic | -2.64 | (0.31) | -2.76 | (0.28) | -2.82 | (0.29) | <.0001 | 0.574 | 0.007 |
| C20:5n3 | Eicosapentaenoic | -2.24 | (0.36) | -2.22 | (0.37) | -2.35 | (0.39) | 0.338 | 0.004 | 0.003 |
| C22:5n3 | Docosapentanoic | -1.43 | (0.20) | -1.35 | (0.20) | -1.23 | (0.18) | <.0001 | 0.012 | <.0001 |
| C22:6n3 | Docosahexaenoic | -0.89 | (0.36) | -1.00 | (0.41) | -0.96 | (0.41) | <.0001 | 0.272 | 0.093 |

P values derived from Wilcoxon signed-rank test comparing fatty acid concentrations measured at ^1^6 weeks restricted to those measured at 6 months, ^2^6 months restricted to those measured at 12 months and ^3^6 weeks restricted to those measured at 12 months. Bonferroni-adjusted level of statistical significance is α=0.05/32 = 0.0016.


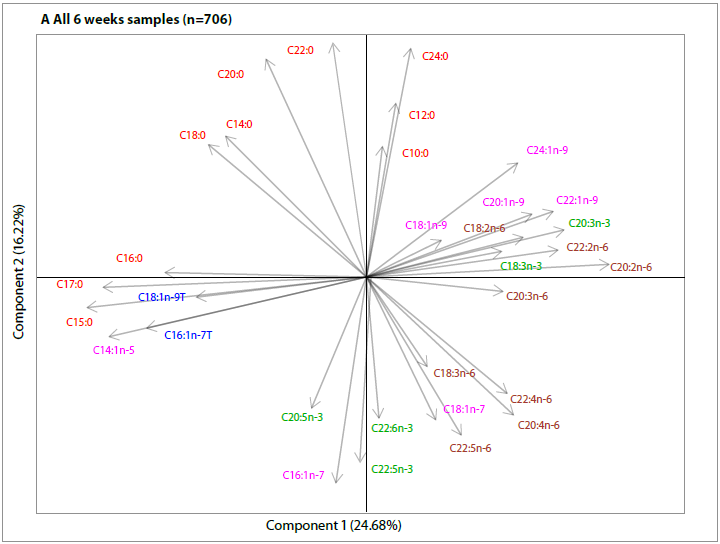

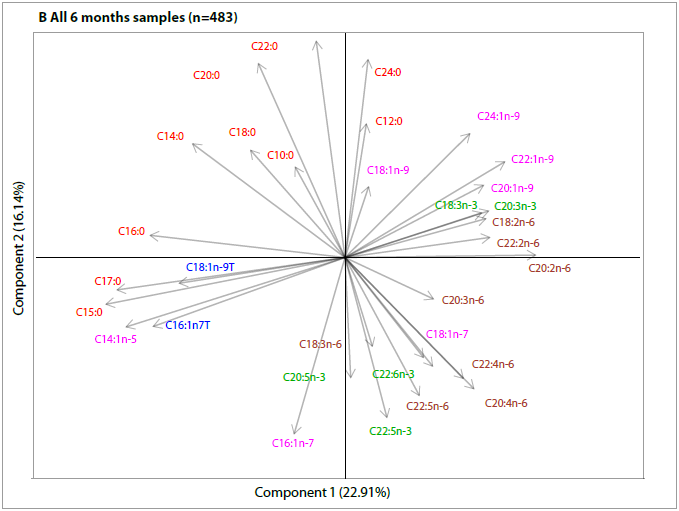

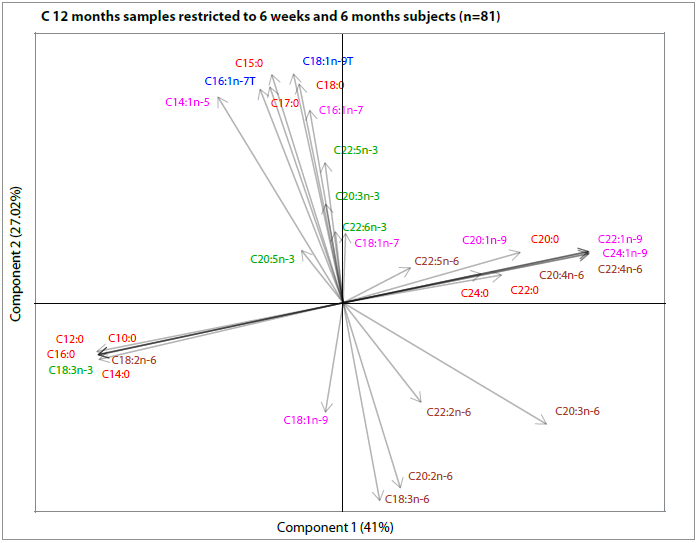

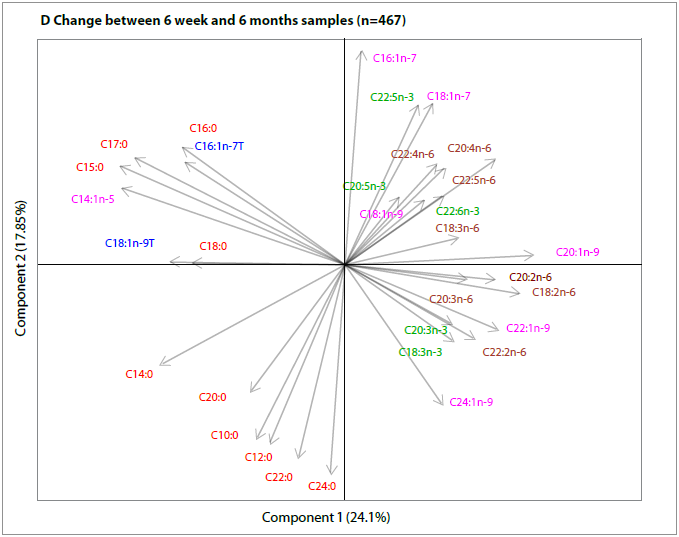

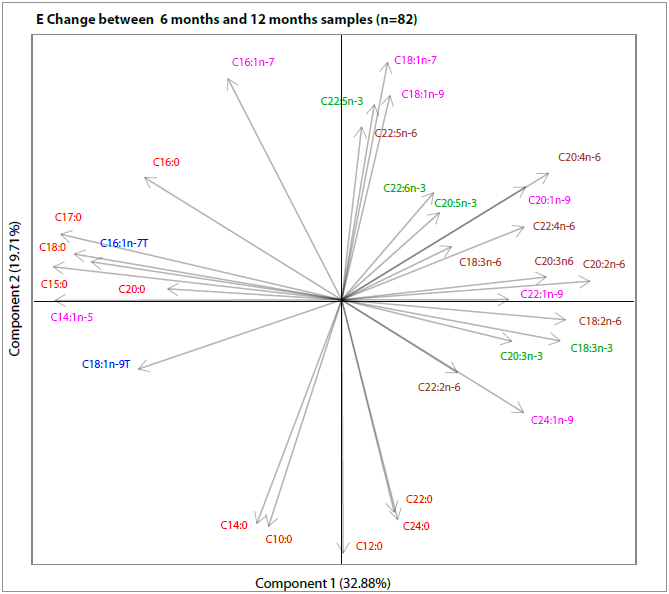

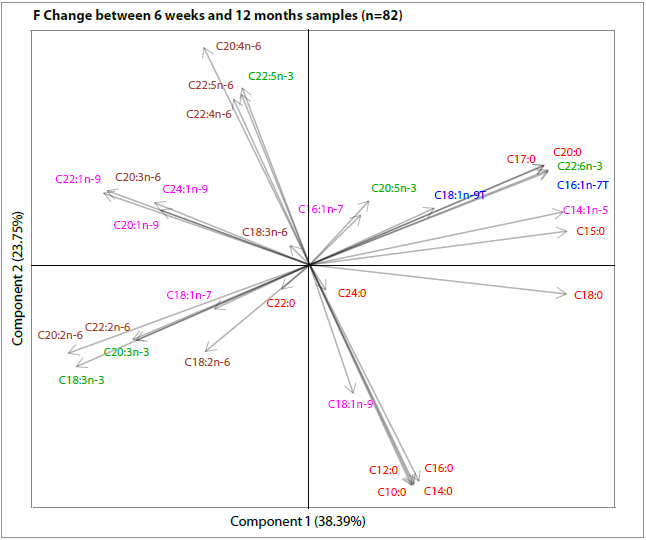


Colour key: Saturated; Trans-; Omega-3; Omega-6; Monounsaturated; Branched-chain

Figure S2: Compositional biplots from principal component analysis (PCA) of selected (32) clr-transformed fatty acid from human milk samples collected at 6 weeks, 6 months and 12 months post-delivery.

| **Fatty acid** | **Common name** | **6 weeks**  **(n=706)** | | **6 months**  **(n=483)** | | **12 months**  **(n = 83)** | | **Pvalue^1^** | **Pvalue^2^** | **Pvalue^3^** |
| --- | --- | --- | --- | --- | --- | --- | --- | --- | --- | --- |
| C8:0 | Caprylic | 0.24 | (0.08) | 0.23 | (0.08) | 0.19 | (0.08) | 0.9737 | 0.3038 | 0.6248 |
| C10:0 | Capric | 1.79 | (0.49) | 1.75 | (0.45) | 1.42 | (0.31) | 0.3572 | 0.8407 | 0.9053 |
| C11:0 | Undecylic | 0.02 | (0.01) | 0.02 | (0.01) | 0.02 | (0.01) | 0.3342 | 0.0575 | 0.0891 |
| C12:0 | Lauric | 5.80 | (1.89) | 6.41 | (1.81) | 6.84 | (1.60) | <.0001 | <.0001 | <.0001 |
| C13:0 | Tridecylic | 0.04 | (0.01) | 0.04 | (0.01) | 0.04 | (0.01) | 0.0258 | 0.2646 | 0.0147 |
| C14:0 | Myristic | 6.43 | (1.65) | 7.41 | (1.78) | 9.29 | (1.90) | <.0001 | <.0001 | <.0001 |
| C15:0 | Pentadecylic | 0.40 | (0.12) | 0.41 | (0.11) | 0.41 | (0.12) | 0.6175 | 0.3214 | 0.867 |
| C16:0 | Palmitic | 23.21 | (2.44) | 22.87 | (2.37) | 23.30 | (2.47) | 0.0006 | 0.9014 | 0.0845 |
| C17:0 | Margaric | 0.32 | (0.07) | 0.32 | (0.06) | 0.33 | (0.07) | 0.6019 | 0.8094 | 0.9266 |
| C18:0 | Stearic | 7.07 | (1.37) | 7.21 | (1.33) | 7.17 | (1.25) | 0.3535 | 0.6511 | 0.3838 |
| C19:0 | Nonadecylic acid | 0.03 | (0.01) | 0.03 | (0.01) | 0.03 | (0.01) | 0.8513 | 0.8263 | 0.8185 |
| C20:0 | Arachidic | 0.21 | (0.04) | 0.21 | (0.04) | 0.21 | (0.06) | 0.0589 | 0.8329 | 0.0128 |
| C22:0 | Behenic | 0.09 | (0.02) | 0.09 | (0.03) | 0.10 | (0.05) | 0.2250 | 0.8433 | 0.0976 |
| C23:0 | Tricosylic | 0.01 | (0.01) | 0.01 | (0.01) | 0.01 | (0.01) | 0.1959 | 0.3463 | 0.7915 |
| C24:0 | Lignoceric | 0.06 | (0.02) | 0.05 | (0.02) | 0.07 | (0.04) | <.0001 | 0.2424 | 0.9947 |
| C12:1n-1 |  | 0.02 | (0.01) | 0.02 | (0.01) | 0.02 | (0.01) | 0.1498 | 0.6779 | 0.8055 |
| C14:1n-5 | Myristoleic | 0.31 | (0.10) | 0.31 | (0.10) | 0.29 | (0.10) | 0.0998 | 0.1258 | 0.2183 |
| C16:1n-7 | Palmitoleic | 2.52 | (0.73) | 2.31 | (0.65) | 2.06 | (0.53) | <.0001 | 0.1914 | 0.0009 |
| C18:1n-7 | Vaccenic | 1.72 | (0.32) | 1.58 | (0.28) | 1.47 | (0.25) | <.0001 | 0.3038 | <.0001 |
| C18:1n-9 | Oleic | 35.15 | (3.36) | 34.64 | (3.15) | 32.63 | (2.93) | 0.0026 | 0.0001 | <.0001 |
| C20:1n-9 | Eicosenoic | 0.44 | (0.09) | 0.39 | (0.09) | 0.39 | (0.12) | <.0001 | 0.7503 | <.0001 |
| C22:1n-9 | Erucic | 0.08 | (0.02) | 0.07 | (0.02) | 0.07 | (0.03) | <.0001 | 0.0502 | 0.0420 |
| C24:1n-9 | Nervonic | 0.06 | (0.03) | 0.05 | (0.02) | 0.05 | (0.03) | <.0001 | 0.036 | 0.8565 |
| C15:0 anteiso | Anteisopentadecylic | 0.12 | (0.08) | 0.13 | (0.05) | 0.13 | (0.05) | 0.2932 | 0.4986 | 0.4693 |
| C16:0 iso | Isopalmitic | 0.09 | (0.03) | 0.09 | (0.03) | 0.09 | (0.03) | 0.6349 | 0.2554 | 0.9493 |
| C18:0i |  | 0.03 | (0.01) | 0.03 | (0.01) | 0.03 | (0.01) | 0.9425 | 0.8709 | 0.9746 |
| C14:1n-5t | Myristelaidic | 0.00 | (0.00) | 0.00 | (0.00) | 0.00 | (0.00) | 0.2221 | 0.3228 | 0.5474 |
| C15:1n-5t |  | 0.00 | (0.00) | 0.00 | (0.00) | 0.00 | (0.00) | 0.0078 | 0.7489 | 0.6679 |
| C16:1n-7t |  | 0.03 | (0.01) | 0.03 | (0.01) | 0.04 | (0.02) | 0.0317 | 0.7965 | 0.6415 |
| C18:1n-9t | Elaidic | 0.45 | (0.32) | 0.49 | (0.40) | 0.57 | (0.30) | 0.5839 | 0.8683 | 0.1858 |
| C18:2n-6tt | Linolelaidic | 0.03 | (0.05) | 0.04 | (0.05) | 0.08 | (0.04) | 0.3871 | 0.071 | 0.7125 |
| C18:2n-6 | Linoleic | 10.33 | (2.79) | 10.18 | (2.63) | 10.20 | (2.71) | 0.7271 | 0.241 | 0.2019 |
| C18:3n-6 | γ-linolenic | 0.12 | (0.04) | 0.10 | (0.03) | 0.07 | (0.03) | <.0001 | <.0001 | <.0001 |
| C20:2n-6 | Eicosadienoic | 0.27 | (0.06) | 0.22 | (0.04) | 0.22 | (0.05) | <.0001 | 0.4185 | <.0001 |
| C20:3n-6 | Dihomo-γ-linolenic | 0.36 | (0.09) | 0.25 | (0.05) | 0.24 | (0.06) | <.0001 | 0.0085 | <.0001 |
| C20:4n-6 | Arachidonic | 0.43 | (0.08) | 0.38 | (0.08) | 0.39 | (0.12) | <.0001 | 0.7150 | 0.0164 |
| C22:2n-6 | Docosadienoic | 0.03 | (0.01) | 0.02 | (0.01) | 0.02 | (0.01) | <.0001 | 0.0854 | 0.0004 |
| C22:4n-6 | Adrenic | 0.09 | (0.02) | 0.07 | (0.02) | 0.08 | (0.03) | <.0001 | 0.0007 | 0.7823 |
| C22:5n-6 | Osbond | 0.04 | (0.01) | 0.03 | (0.01) | 0.03 | (0.01) | <.0001 | 0.9573 | 0.0004 |
| C18:3n-3 | α -linoleic | 0.93 | (0.39) | 0.97 | (0.40) | 0.86 | (0.31) | 0.3614 | 0.0571 | 0.1686 |
| C20:3n-3 | Dihomo-α-linoleic | 0.04 | (0.01) | 0.04 | (0.01) | 0.03 | (0.01) | <.0001 | 0.7088 | 0.0002 |
| C20:4n-3 |  | 0.09 | (0.04) | 0.06 | (0.02) | 0.05 | (0.03) | <.0001 | 0.0002 | <.0001 |
| C20:5n-3 | Eicosapentaenoic | 0.07 | (0.03) | 0.07 | (0.04) | 0.06 | (0.03) | <.0001 | 0.0084 | <.0001 |
| C22:5n-3 | Docosapentanoic | 0.15 | (0.04) | 0.15 | (0.04) | 0.16 | (0.04) | 0.1474 | 0.0764 | 0.2800 |
| C22:6n-3 | Docosahexaenoic | 0.27 | (0.13) | 0.23 | (0.12) | 0.23 | (0.13) | <.0001 | 0.4033 | 0.0079 |

Table S4: Means and standard deviation for individual fatty acids from human milk samples collected at 6 weeks, 6 months and 12 months post-delivery (%weight of total fatty acids)

P values derived from Wilcoxon signed-rank test comparing fatty acid concentrations between ^1^6weeks and 6 months, ^2^6months and 12months, ^3^6weeks and 12months. Bonferroni-adjusted level of statistical significance is α=0.05/45 = 0.0011.
